# Preemptive Treatment of De Novo Donor Specific Anti-HLA Antibodies With IVIG Monotherapy after Lung Transplantation

**DOI:** 10.3389/ti.2024.13431

**Published:** 2024-09-19

**Authors:** Jennifer K. McDermott, Skye J. Castaneda, Sarah M. Mietz, Cameron K. Lawson, John A. Gerlach, Ryan J. Hadley, Gayathri Sathiyamoorthy, Sheila Krishnan, Edward T. Murphy, Reda E. Girgis

**Affiliations:** ^1^ Richard DeVos Heart and Lung Transplant Program, Corewell Health and Michigan State University College of Human Medicine, Grand Rapids, MI, United States; ^2^ Biomedical Laboratory Diagnostics Program, Department of Medicine, Michigan State University College of Human Medicine, East Lansing, MI, United States

**Keywords:** DSA *de novo*, HLA antibodies, lung transplant, IVIG, CLAD-free survival

Dear Editors,

Development of *de novo* donor specific anti-HLA antibodies (dnDSA) following lung transplantation (LTX) is common and increases the risk for chronic lung allograft dysfunction (CLAD) and death [1–3]. Optimal management of asymptomatic dnDSA, in the absence of clinical antibody-mediated rejection or allograft dysfunction, is unclear. The approach varies among transplant centers. Some do not initiate specific therapy, while others pre-emptively treat with regimens that typically include high-dose intravenous immunoglobulin (IVIg) [4–6]. A recent retrospective multi-center report found improved CLAD-free survival among 30 LTX recipients with asymptomatic dnDSA who received preemptive therapy compared with 115 controls who did not receive antibody-targeted therapy or were only treated once signs of AMR developed. Various antibody reduction strategies were employed, including IVIg alone or in combination with rituximab, plasmapheresis, bortezomib, tocilizumab, and/or methylprednisolone [6].

IVIg has multiple immunomodulatory effects including neutralization of pathogenic IgG, inhibition of cytokine gene activation and activity, interaction with antigen-presenting cells to suppress T-cell activation, expansion of regulatory T-cells and inhibition of complement activity [7, 8]. We routinely administer pre-emptive high dose IVIg monotherapy for asymptomatic dnDSA in an attempt to reduce the strength of the antibody and improve long-term outcomes. The aim of this study is to review our experience with this approach.

Institutional review board approval was obtained. Data was collected from our prospective LTX registry. HLA antibody by Luminex single antigen bead assay was routinely obtained at post-op day 14, at 1-, 3-, 6-, 9-, 12- and 18-month post-transplant in conjunction with surveillance bronchoscopy with biopsy, yearly thereafter, and at any time for clinical indication. During the study period from 2/2013 and 3/2021, 230 lung transplants were performed. Sixty-three recipients (27%) developed dnDSA. We excluded 16 with a sum mean fluorescence intensity (MFI) < 3,000, 7 with clinical AMR at the time of first detection of dnDSA, 6 with non-HLA indication for IVIg and 2 re-transplants. The remaining 32 recipients with sum mean MFI ≥3,000 and no evidence of allograft dysfunction or AMR were treated with IVIg monotherapy at 2 gm/kg followed by 1 gm/kg monthly, for a minimum of 3 months or until clearance (sum MFI <1,000) up to 6 months. The cohort was predominantly Caucasian (84%), bilateral LTX in 72%, 53% male with a median age of 62 (IQR:59, 67). Transplant indication was idiopathic pulmonary fibrosis in 15 (47%) and chronic obstructive pulmonary disease in 11 (34%). Median time to development of dnDSA was 33 days (IQR:18, 143). The immunodominant DSA was frequently class II (N = 26, 81%), most commonly DQ (N = 24, 75%). Median dnDSA sum MFI before treatment was 4782 (IQR: 2,937, 7,490) and decreased by a median of 2,993 (IQR: 2051, 6,358) after the initial course of IVIg; P < 0.0001. Eighteen (56%) achieved DSA clearance. (Figure 1) Among the 14 patients without clearing of DSA, IVIg was associated with a significant reduction in DSA strength (median sum MFI at baseline: 5,462 [IQR: 3,830, 9,770] vs. 2,714 [1899, 370] after treatment, P = 0.017). Additional IVIg was given to 8 patients with persistent DSA and 5 with recurrence after clearing with the first course. At last follow-up at a median of 1,330 days post-transplant (IQR: 861, 1910), clearance of DSA was present in 23 (72%).

**FIGURE 1:**
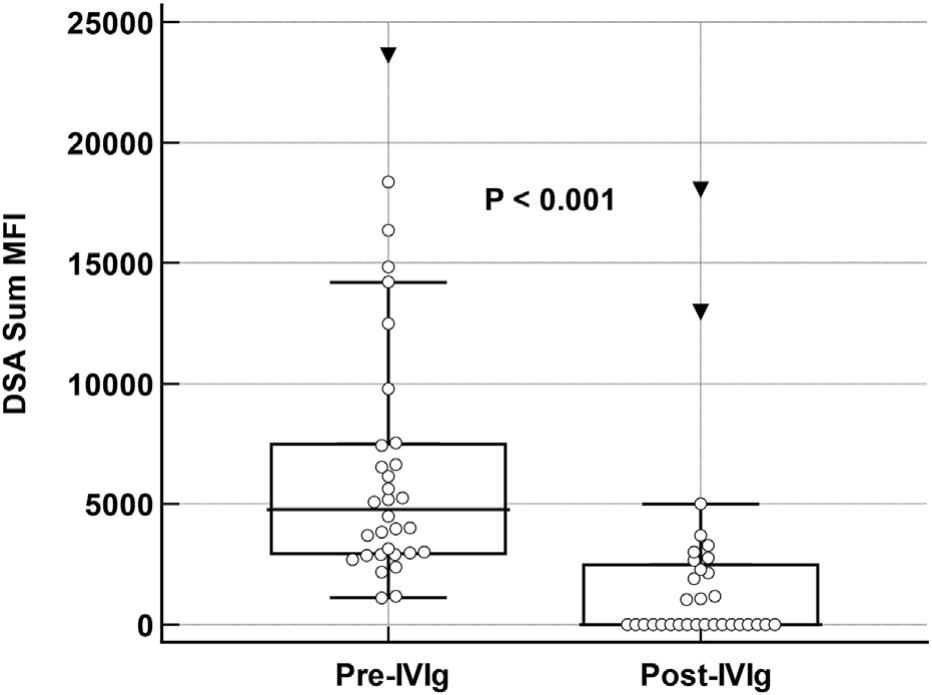
Changes in DSA sum MFI pre and post Therapy with IVIg. Box and whisker plot demonstrating change in donor-specific antibody sum mean fluorescent intensity values at baseline and after intravenous-immune globulin monotherapy in 32 lung transplant recipients. Analysis was performed with Wilcoxon rank sum test for paired samples.

During the observation period, CLAD had developed in 11 subjects and 10 died. CLAD-free survival at 2- and 4-year post-transplant were 79% and 62%, respectively. In comparison, CLAD-free survival in a concurrent cohort of 128 recipients with no DSA was 92% and 77% at 2- and 4-year, respectively (P = 0.023 by log-rank comparison of Kaplan-Meir survival curves). We observed a trend towards a lower incidence of subsequent acute cellular rejection (61% vs. 93%; P = 0.05) and AMR (6% vs. 36%; P = 0.06) in those who cleared DSA compared with those with persistent DSA after IVIg, but similar CLAD incidence and survival.

A large body of data has demonstrated a strong association between dnDSA, allograft rejection, and subsequent CLAD and mortality in lung transplant recipients. The association of stronger antibody (higher MFI) and complement fixing ability of dnDSA with worse outcomes and its detection prior to allograft dysfunction suggests that these antibodies may be involved in the pathogenesis of injury, rather than simply a marker of disease [1, 9]. In this series of asymptomatic dnDSA lung transplant recipients, we observed significant reductions in dnDSA MFI following preemptive IVIg monotherapy with clearing in over half.

In the absence of a control group, it is not possible to draw firm conclusions regarding the efficacy of this approach. Spontaneous clearing of dnDSA has been observed in up to one-third of cases [1, 10] and decreasing MFI in over half [9]. However, the definition of positive DSA in these reports was lower than our threshold MFI of 3,000. Clinical outcome, in terms of CLAD-free survival, of this cohort was comparable to other studies where additional measures were employed, such as plasma exchange and B-cell depleting therapy [4–6]. While CLAD-free survival was lower than our concurrent DSA-negative group, it was similar to the DSA-negative cohort of Keller et al (2-year CLAD-free survival of 70%) and markedly better than the no pre-emptive therapy DSA-positive group in that study (2-year CLAD-free survival of 42%) [6]. However, caution needs to be exercised in comparing these survival rates as our group was small and the populations different. Importantly, CLAD-free survival was similar among subjects who cleared DSA with pre-emptive therapy vs. those with persistent DSA in the multicenter study, similar to our observation. This could reflect a reduction in the strength of DSA and/or other immunomodulatory effects of treatment.

Taken together with the Keller study [6], our findings suggest that preemptive therapy for asymptomatic dnDSA may improve long-term outcomes in this high-risk group. The use of IVIg monotherapy appears to yield similar results to those of combination strategies that are more cumbersome and associated with greater potential for complications. However, prospective, randomized controlled trials are required to definitively assess the efficacy of preemptive therapy and the optimal regimen.

## Data Availability

The raw data supporting the conclusions of this article will be made available by the authors, without undue reservation.

## Abbreviations

ACR, acute cellular rejection; AMR, antibody-mediated rejection; CLAD, chronic lung allograft dysfunction; dnDSA, *de novo* donor specific anti-HLA antibodies; HLA, human leukocyte antigen; iDSA, immunodominant DSA; IQR, interquartile range; IVIg, intravenous immune globulin, MFI, mean fluorescence intensity.
